# Clinical study of stent forming for symptomatic internal carotid artery stenosis

**DOI:** 10.1097/MD.0000000000020637

**Published:** 2020-06-19

**Authors:** Yiqin Zhang, Wen’an Wang, Qi Fang

**Affiliations:** a The First Affiliated Hospital of Soochow University, Suzhou, Jiangsu; b Chongming Branch, Xinhua Hospital, Shanghai Jiao Tong University School of Medicine, Shanghai; c The First Affiliated Hospital of Soochow University, Suzhou, Jiangsu, China.

**Keywords:** choice of treatment strategy, clinical application and efficacy, stenting, symptomatic carotid stenosis

## Abstract

Stroke is one of the diseases that seriously threaten the survival and health of human beings. In Europe and the United States, stroke is the second leading cause of death after heart disease and tumors.^[1]^ Stroke is also one of the fatal diseases in Asian countries. On average, about 1.5 million new stroke patients are added each year in China, and the incidence of stroke is increasing year by year. About 80% of all stroke patients are ischemic stroke, and symptomatic internal carotid atherosclerotic stenosis is another important cause of ischemic stroke. Ipsilateral carotid stenosis ≥50% increases the incidence of transient cerebral ischemia and stroke in the carotid artery region by 10% to 15%, and is also closely related to the recurrence of acute and long-term stroke in patients.^[2]^ Therefore, the clinical application and efficacy of carotid stenting in patients with symptomatic internal carotid artery stenosis are analyzed and evaluated to provide a basis for the selection of clinical treatment options.

The clinical data of patients with symptomatic carotid stenosis who underwent carotid stenting and the clinical data of conservative treatment of patients with symptomatic carotid stenosis were retrospectively analyzed, and the carotid stenosis rate, symptoms, and National Institute of Health stroke scale where compared before and after surgery. And activities of daily living score. Because the control group treatment method in this article is completely free for patients to choose, and belongs to a retrospective analysis, the results suggest that it can provide a high-quality treatment approach for the treatment of patients with symptomatic carotid stenosis, without causing any harm to the patient, So no ethical approval is needed, and no patient informed consent is required.

In recent years, with the continuous advancement of science and technology and new stent materials, intravascular interventional technology has developed rapidly. In continuous clinical practice and research, the safety and effectiveness of stent technology have also been gradually improved. Arterial stenting has gradually become an important method for the treatment of atherosclerotic carotid stenosis. This technique can not only improve the symptoms and prognosis of patients with symptomatic internal carotid stenosis, but also prevent the occurrence of ischemic events. The promotion of this technology has the effect of reducing disability and mortality.

The alternative therapy of drug therapy, namely arterial stent implantation, has become a new way to treat atherosclerotic stroke. This treatment technology can quickly relieve the abnormal hemodynamics of distal blood vessels caused by arterial stenosis, which is ischemic. Cerebrovascular disease provides new ideas for treatment. Carotid angioplasty and stenting for symptomatic internal carotid stenosis under a distal cerebral protection device is a safe and effective treatment.

## 1 Introduction

The definition of symptomatic internal carotid artery stenosis is currently clinically interpreted as: patients with infarcts associated with narrowed blood vessels within the last 6 months, manifestations include difficulty vocalization, numbness and muscle weakness in the limbs and face, drinking choking, Vision loss, temporary darkness. Clinical studies have shown that symptomatic carotid stenosis is closely related to the risk of recurrent ischemic stroke, such as cerebral infarction and transient cerebral ischemia (TIA) patients. Symptomatic atherosclerotic stenosis is one of the important risk factors for ischemic stroke. With the development of imaging in recent years, the following effective examination methods: magnetic resonance angiography (MRA), computed tomographic angiography (CTA), and digital subtraction angiography (DSA) have been widely used to judge carotid stenosis. There are 3 clinical options available for the current treatment of symptomatic internal carotid artery stenosis, including medical, surgical, and vascular interventional therapy. Medical treatment is mainly through changing bad living habits, such as smoking cessation, low-salt, low-fat diet, among others, and the combined use of drugs to control basic diseases such as blood sugar, blood pressure, blood lipids, and then long-term oral aspirin, clopidogrel, and other antiplatelet drugs to delay the carotid Stenosis progression, a treatment that reduces the symptoms associated with stenosis. Surgical treatment is mainly by peeling the carotid intima to achieve the goals of removing thickened intima and atherosclerotic plaques, releasing vascular stenosis, and restoring normal blood supply to the brain. Vascular interventional treatment is mainly by implanting a suitable stent at the diseased blood vessel, thereby expanding the carotid stenosis and improving blood supply to the brain.^[3–6]^

Patients with symptomatic arterial stenosis are selected as follows:

1. Indications: Patients with clinical prodromal symptoms or clinical symptoms of stroke, patients with mild carotid artery stenosis on imaging examination; patients with ischemic symptoms, 50% ≤ vascular diameter stenosis <99%; unstable plaques.
2. Contraindications: The degree of stenosis of blood vessel diameter is ≥99%; the arterial blood vessel is severely tortuous and deformed; unstable emboli are formed in the carotid artery; the blood vessel wall is severely calcified and difficult to expand; those with severe allergy to contrast agents; those with severe heart, liver and kidney dysfunction.

This study retrospectively analyzed the clinical data of 128 patients with symptomatic carotid stenosis who underwent carotid stenting in Shanghai, China, from October 2016 to October 2018, and compared the carotid stenosis rate, symptoms, NIHSS score, and ADL before and after surgery.

### 1.1 Scoring situation

The clinical data of 132 patients with symptomatic carotid stenosis who were conservatively treated were retrospectively analyzed. They were divided into drug treatment group and endovascular stent implantation group. The 2 groups were divided according to sex, age, hypertension, hyperlipidemia, diabetes, and smoking. There were no significant differences in the risk factors of cerebrovascular disease in history. The average age of patients receiving carotid angioplasty and stenting (CAS) in this study was 68.7 years, and the oldest was 86 years’ old. They were more complicated with medical diseases such as hypertension, diabetes, hyperlipidemia, and severe and extremely severe stenosis accounted for 59.7%. Most of them combined with medical diseases such as hypertension, diabetes, hyperlipidemia, and severe and extremely severe stenosis accounted for 59.7%. Although some patients suffered complications such as heart rate reduction and blood pressure reduction during operation, they returned to normal after symptomatic treatment; Statistically follow up the occurrence of complications after stenting in patients.

### 1.2 Results

The preoperative DSA angiography showed that the carotid artery stenosis rate was 74.6% ± 12.9%. The stent was implanted under a brain protection device, and no stroke or death occurred during the operation. DSA angiography showed the average residual stenosis immediately after the operation. It was (12.4% ± 7.7%), compared with *P* < .05 compared with before surgery. During the post-hospitalization period, 2 cases of stroke occurred, and a small number of patients developed hypotension, mild headache, nausea, and angina; however, no cerebral hemorrhage and stroke-related death occurred. During an average follow-up of (12.6 ± 5.6) months, 79 patients (52.8%) had symptoms completely disappeared before surgery, 25 (47.2%) had neurological impairment of varying degrees, and TIA occurred in 5 (7.5%). Ipsilateral stroke occurred in 4 (3.0%), angina pectoris occurred in 6 (4.5%), and no stroke-related death occurred; 64 (41.8%) underwent DSA angiography and 42 (32.8%) underwent CTA. On re-examination, 22 patients (25.4%) underwent ultrasound re-examination, and none of them had a stenosis rate exceeding 50%. The 6-month postoperative NIHSS score (0.75 ± 0.42) was compared with the preoperative NIHSS score (2.96 ± 1.45), *P* < .05 (Table 1).

**Table 1:**
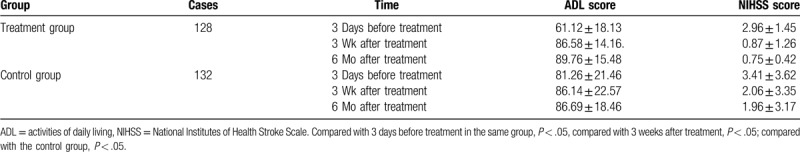
Comparison of ADL and NIHSS scores between treatment group and control group at different time points.

### 1.3 Statistical analysis

The SPSS18.0 statistical software package is used for the analysis. The quantitative data are described by mean ± standard deviation (X ± S). Or the composition ratio is described. The data were first tested for normality. For a sample that fits the normal distribution, a 2-sample *t* test was used. For data that were significantly skewed, a rank-sum test was used. The count data were tested by a *χ*^2^ test. The difference was statistically significant with *P* < .05.

## 2 Result

Perioperative and follow-up periods only occurred in 5 cases of TIA, 4 cases of stroke, and no stroke-related deaths. This result is similar to some foreign literature reports. Therefore, it is believed that CAS is safe and feasible for the treatment of symptomatic carotid stenosis, and the incidence of complications is low. According to the patient's clinical manifestations, carotid artery stenosis, etc., under the brain protection device, skilled use of carotid stenting to treat symptomatic internal carotid artery stenosis, timely and effective treatment of various complications, is expected to obtain satisfactory results, but the long-term clinical efficacy of this technology needs further observation. The effect needs further observation.

At 6 months after CAS, the NJHSS score was significantly lower than before CAS, and there were significant differences in statistical tests, suggesting that CAS can be beneficial to the recovery of cognitive function, which is consistent with some domestic and foreign research reports. The causes of cognitive dysfunction caused by carotid stenosis may be related to chronic hypoperfusion, lacunar infarction, white matter damage, and stroke. After CAS, carotid stenosis is basically relieved, intracranial blood supply is increased, hypoperfusion state is relieved or disappeared, cerebral blood flow is gradually restored, metabolism of various substances is gradually restored to normal, and harmful substances such as ischemia and excitatory amino acids are gradually eliminated. As a result, further damage to neurons is reduced, the function of the damaged part is gradually compensated by normal brain tissue, and cognitive function is improved.^[7–13]^ Some studies have suggested that moderate to severe internal carotid artery stenosis is an independent risk factor for multiple lacunar cerebral infarction. CAS improves cerebral blood flow perfusion, reduces the occurrence of lacunar cerebral infarction, and can prevent further deterioration of cognitive function. This retrospective analysis of this group of cases has initially confirmed that CAS for symptomatic internal carotid artery stenosis under CPD is a safe and effective treatment method. By fully understanding the risk factors related to complications, timely and effective treatment can be allowed patients to obtain satisfactory results.^[14–19]^

## 3 Discussion

The etiology and pathogenesis of symptomatic internal carotid artery stenosis are related to the following factors: atherosclerosis is an important cause of carotid artery stenosis, in addition to vasculitis, neck trauma, arterial dissection, radiation vasculitis, and compressive lesions outside the artery; fibromuscular dysplasia, congenital vascular malformations, syphilis, among others, can also cause narrowing of blood vessels. Among them, related risk factors for atherosclerosis include: hypertension, hyperlipidemia, diabetes, alcoholism, smoking, lack of exercise, abdominal obesity, and high mental stress. In patients with carotid artery stenosis, when the stenosis rate exceeds 50%, the blood flow velocity increases, and when the stenosis exceeds 70%, the blood flow velocity will increase significantly. The increase of blood flow velocity will obviously increase the shear stress of vascular endothelial cells and cause endothelial cell damage. Inducing platelet aggregation and thrombosis, it is easy to cause adherent thrombus to fall off and cause cerebral infarction.^[20]^

The diagnosis of symptomatic carotid artery stenosis is based on prodromal symptoms such as limb numbness, inability to move, headache, dizziness, and drowsiness; symptoms or signs related to brain damage such as hemiplegia, sensory disturbance, aphasia, and blindness. Skull computed tomography is normal in the early stage, and low-density foci can appear after 24 to 48 hours. B ultrasound, computed tomography angiography, magnetic resonance angiography, and cerebral angiography suggest carotid stenosis.

### 3.1 Judgment method of carotid artery stenosis

The degree of stenosis of the diameter of the blood vessel is measured with reference to the standards established by NASCET, and the formula is: vascular diameter stenosis rate = (l-inner diameter of the blood vessel at the lesion/inner diameter of the distal normal blood vessel at the lesion) × 100%. Medical treatment mainly uses drugs to reduce the symptoms of cerebral ischemia, delay the process of vascular stenosis, and thereby reduce the risk of stroke. It is mainly suitable for conservative treatment of patients with mild carotid artery stenosis. Carotid endarterectomy can reduce stroke and TIA. Today, CEA has been widely used in the treatment of carotid stenosis and has become the “criterion standard.” This group of studies showed that the rate of carotid diameter stenosis after CAS confirmed by DSA decreased from 74.6% ± 12.9% before stent implantation to 12.4% ± 7.7% after stent implantation (*P* < .05), and the degree of vascular stenosis was significant. reduce. Of the 128 patients with symptomatic carotid artery stenosis during the postoperative follow-up period, 79 clinical symptoms completely disappeared, and neurological and cognitive functions gradually improved during the follow-up period, indicating that after the internal carotid artery stenosis was relieved by CAS, cerebral blood flow was significantly restored. The blood perfusion in the hypoxic area of the brain tissue is effectively improved, which helps to improve the clinical symptoms of patients.^[12]^ However, the stenosed blood vessels must not be excessively pursued during surgery for anatomical complete recovery. The reasons are as follows:

1. Research suggests that as long as the cerebral blood flow can be restored to more than half of the normal value after surgery, brain nerve cells can maintain normal electrophysiology activity.
2. Excessive expansion will cause the stent to cut and tear plaques, increasing the risk of cerebral embolism; and excessive squeezing of lipid components into the stent may also cause acute occlusion of blood vessels.
3. Excessive expansion can destroy the structure of the blood vessel wall, causing excessive proliferation of endothelial and smooth muscle cells, and causing the occurrence of intravascular restenosis.
4. For severely narrowed blood vessels with severe calcification, excessive dilation can cause blood vessel tearing, with catastrophic consequences.
5. Cerebrovascular self-regulation is limited. Vascular stenosis in patients with overdilation will increase the probability of cerebral hyperperfusion syndrome and cerebral hemorrhage, especially in the treatment of high-risk cases of severe vascular stenosis, delayed cerebral angiography, poor intracranial perfusion, insufficient compensation or lack Avoid excessive expansion.

This study suggests that the NIHSS score in the second half of CAS is significantly lower than before CAS, and there are significant differences in statistical tests, indicating that after CAS improves carotid stenosis and restores intracranial blood flow, nerve function will gradually recover: 6 before and after surgery The monthly comparison of neurological function score (ADL score) was significantly improved, and the difference was statistically significant (*P* < .01). However, the clinical symptoms of only a few patients in the control group improved, and there was no significant difference in ADL and NIHSS scores before and after conservative treatment. The comparison of the ADL score between the treatment group and the control group was statistically significant (*P* < .05), indicating that the clinical effect of endovascular stentplasty for symptomatic carotid stenosis is better than that of drug treatment. After clinical practice, we found that under the premise of strictly grasping the indications, active CAS treatment of symptomatic carotid stenosis can effectively relieve the symptoms of cerebral ischemia and promote the recovery of neurological function in patients,^[21,22]^ which may be related to the following (the reasons are related)

1. Because of the ischemic and hypoxic cerebral infarction, there is a hypofunctional area. When the intracranial blood supply is improved after the stenosis is relieved, the brain cells in this area will gradually become active, thereby partially compensating the features.
2. After the carotid artery stenosis is relieved, the blood oxygen content in the brain tissue can be increased, the hypoxic state of the brain cells is improved, the free radical generated by hypoxia can continue to destroy the nerve cells, and promote the recovery of reversible brain tissue damage.
3. The improvement of carotid stenosis may cause the establishment of partial collateral circulation in the brain, improve the microcirculation state of the brain, and increase the supply of oxygen and nutrients.
4. For some patients with unstable plaques, stent placement can play a role in stabilizing plaques, reducing emboli detachment, effectively preventing the recurrence of stroke, and preventing further deterioration of nerve function.
5. After the vascular stenosis of the neck is relieved, the local brain tissue hypoxia stimulation caused by it disappears, and the cerebral blood vessel system with the disorder of the hyperactive sympathetic nervous system function and self-regulating function can gradually return to normal.
6. Can delay or even reverse the evolution of hypoperfusion zone to infarction to some extent.

Common complications of CAS and related risk factors are: intraoperative and perioperative patients with reduced blood pressure and heart rate; intravascular vasospasm; atheromatous plaques and thrombosis; perioperative ischemic stroke; hyperperfusion symptoms; residual stenosis after stent expansion, and restenosis, displacement, deformation, and stent dislocation; arterial puncture-related complications such as hematoma and subcutaneous congestion. Cerebral ischemic event is one of the serious complications of CAS. Patients may have clinical manifestations such as loss of motor function, aphasia, and sensory disturbances, which are mainly related to the following factors: preoperative application of antiplatelet drug dose and insufficient time; plaque shedding during interventional device operation; balloon dilation, stent release Plaque squeezing and cutting leads to plaque shedding; gas embolism due to improper operation; insufficient heparinized dose during operation, postoperative use of antiplatelet, anticoagulant drug dose, insufficient time, or insensitivity to antiplatelet drugs, and stent thrombosis occurred. The use of brain protection devices during the operation can prevent most of the emboli from falling into the skull. However, postoperative neurological examination and imaging examination should be paid attention to. If strokes are found, they should be treated in time. Some studies have found that patients older than 75 years with >2 risk factors for cerebrovascular disease, a history of multiple cerebral infarctions before surgery, and a lack of systemic antiplatelet medication after surgery are prone to undergo ischemic cerebrovascular disease after surgery. It is very important for patients to monitor closely after operation and to apply anticoagulation and antiplatelet therapy regularly. The incidence of stent thrombosis is low, but the mortality rate is high. Therefore, taking bolivive and aspirin antiplatelet aggregation drugs 3 to 5 days before surgery can prevent platelets from accumulating on the plaque. Reasonable application of heparin according to the patient's weight during the operation can effectively prevent thrombosis. After the stent is implanted, it should be reviewed by contrast imaging. If thrombosis is found, thrombolytic treatment should be performed in time. Cerebral hyperperfusion syndrome often occurs in patients with severe internal carotid artery stenosis after CAS. Mainly due to vascular stenosis, the blood supply in the corresponding cerebral blood supply area is insufficient for a long time. Intracranial small blood vessels are dilated due to long-term reflex, which causes the loss of self-regulation ability. Immediately after the stent implantation, a large amount of high-speed blood flow poured into the dilated parenchymal blood vessels, resulting in the occurrence of hyperperfusion syndrome.^[23]^ It can be divided into 2 cases of cerebral edema type and cerebral hemorrhage type. The main symptoms of cerebral edema are headache, nausea, and even epilepsy and coma. Cerebral hemorrhage is mostly caused by the rupture of the blood-brain barrier caused by an increase in cerebral perfusion pressure. It mainly occurs in patients with severe carotid stenosis and poor lateral branch compensation. McCarthy et al reported bleeding in 2 of 81 severely narrowed cases. Cerebral arterial compliance in the elderly has decreased, and vascular self-regulation ability due to long-term ischemia has also significantly decreased. Blood flow in the cerebral ischemic area suddenly increased after stent implantation. In addition, antiplatelet and anticoagulation were routinely applied during perioperative period. Drugs, once bleeding occurs, will be difficult to control with disastrous consequences. Li Baomin and other scholars have a clinical study on whether blood pressure and heart rate control are performed during CAS surgery. The blood pressure was maintained at the lower limit of normal values. The heart rate was controlled at 55 beats per minute during surgery. The heart rate was controlled at 85 beats per minute during balloon expansion.

### 3.2 Results

The incidence of complications was significantly lower in the control group than in the uncontrolled blood pressure group, and the long-term follow-up was satisfactory.^[24]^ In the absence of heart, brain, and kidney ischemia, the patient's blood pressure was strictly controlled to <140/90 mmHg during surgery, and blood pressure was controlled to 120/80 mmHg after surgery, which can reduce the occurrence of cerebral hemorrhage during perioperative period. Therefore, strict blood pressure control during and after CAS is an important measure to effectively prevent cerebral hyperperfusion syndrome.

Intra-stent stenosis after CAS is one of the important complications affecting its long-term efficacy. According to Davis and other studies, restenosis after intravascular stent implantation is usually related to thrombosis, acute inflammatory response, cell aggregation, and extracellular matrix. Intimal cells gather around the scaffold, hyperproliferate, and are associated with foreign body rejection. Bonati et al showed that the 5-year severe restenosis rate was significantly higher in the CAS group than in the CEA group, but significantly lower than that of balloon dilatation alone. Patients with recurrent severe stenosis also had a higher risk of nonsurgical stroke and TIA. This group of patients underwent imaging follow-up. No patients with a stenosis rate of >50% were found, which may be related to regular antiplatelet and lipid-lowering drugs. With the development and application of many new drug-coated stents and biological stents, it will help to further reduce the long-term restenosis rate after CAS.^[25]^

## 4 Conclusion

In the past, the treatment of symptomatic internal arterial stenosis was mostly treated with drugs, but clinically found that patients with drug treatment have a high rate of stroke recurrence. Some studies have shown that up to 12% of patients with ischemic stroke who receive sufficient aspirin treatment. The annual recurrence rate of stroke and vascular stent placement can improve vascular endothelial damage and improve hemodynamics, which better solves such problems. Intravascular stentplasty is minimally invasive, safe, has few complications, and is easy to operate. While improving cerebral blood flow, it can also prevent thrombus formation and shedding. A large number of clinical studies have shown that simple drugs are not effective in treating patients with arterial stenosis, especially in patients with severe stenosis, and the recurrence rate of stroke is higher. Carotid angioplasty and stent implantation have become more and more widely used in patients with carotid stenosis. In the past, it was mostly used for the treatment of patients with moderate to severe carotid stenosis, especially those at high risk who are not suitable for endarterectomy. Clinical studies confirm that the safety and effectiveness of CAS are not inferior to crotid endarterectomy and angioplasty (CEA). CAS can be used as an alternative to CEA. Many research results since SAPPHIRE have shown that the incidence of stroke or death of patients after CAS has gradually decreased, and safety and efficacy have been improved. This may benefit from the improvement of carotid stent in recent years and the maturation of CAS operation technology, and routine use of distal brain protection devices. CAS is minimally invasive and has fewer complications. It is an effective method for treating carotid stenosis and has been gradually accepted by patients and doctors. For patients with mild carotid stenosis after imaging examination, if there are clinical prodromal symptoms or clinical symptoms of stroke, to obtain better clinical results, the clinical symptoms, vascular stenosis rate and lesion site, vascular conditions, and other foundations should be taken according to the patient's clinical symptoms. Individualized treatment options for the wishes of the disease, patients, and family members to truly reduce the risk of surgery can benefit patients, improve symptoms and prognosis related to stenosis, and prevent the occurrence of ischemic events, reduce the disability rate, and the role of case fatality reduces social burden. With the extensive clinical application of brain protection devices, the development of special stent, balloon and guide wire for carotid stenosis, the research on the etiology of stent restenosis, and the improvement of perioperative intervention methods, and the continuous improvement of intravascular operative technology, CAS, as one of the methods to treat carotid stenosis, will further reduce the incidence of perioperative stroke, death, and other complications and adverse events.^[26]^ It is expected that the short-term and long-term effects will continue to improve. CAS will be used in the carotid artery. The treatment of patients with stenosis and the prevention of stroke play a more important role. According to the patient's clinical manifestations, carotid artery stenosis, etc., under the brain protection device, skilled use of carotid artery stenting to treat symptomatic internal carotid artery stenosis, timely and effective treatment of various complications, is expected to obtain satisfactory clinical efficacy, But the long-term clinical efficacy needs further observation. CAS and CEA are two treatments for carotid artery stenosis. For a long time, their efficacy and safety have been the focus of research. At present, CEA is still the “gold standard” and the status is unshakable. However, CAS is minimally invasive and has fewer complications. It is an effective method for treating carotid artery stenosis and is gradually accepted by the majority of patients and doctors. Many large-scale clinical trial studies have gradually confirmed that CAS is not inferior to CEA. In order to obtain better clinical efficacy, patients should be conducted according to the patient's clinical symptoms, vascular stenosis rate and lesion location, vascular conditions, whether there are other underlying diseases, patients and their families Individualized choices to truly reduce surgical risks and benefit patients. With the wide application of brain protection devices, the development of special stents, balloons and guide wires for carotid artery stenosis, the research on the causes of stent restenosis and the improvement of perioperative intervention methods, as well as the continuous improvement of physicians’ intravascular operation techniques The treatment of arterial stenosis will further reduce the incidence of perioperative stroke, death and other complications and adverse events, and improve the short-term and long-term efficacy. CAS will play a more important role in the treatment of patients with carotid artery stenosis and stroke prevention effect. However, the long-term clinical effect needs further observation.

## Author contributions

Yiqin Zhang is responsible for the specific writing and modification of the article, also responsible for the collection and collation of article information. Qi Fang is responsible for the initial written suggestions and specific ideas of the article, and Wenan Wang is responsible for guiding the translation of articles in foreign languages.
